# Risky decision-making is associated with residential choice in healthy older adults

**DOI:** 10.3389/fpsyg.2015.01192

**Published:** 2015-08-11

**Authors:** Kendra L. Seaman, Chelsea M. Stillman, Darlene V. Howard, James H. Howard

**Affiliations:** ^1^Department of Psychology, The Catholic University of AmericaWashington, DC, USA; ^2^Department of Psychology, Georgetown UniversityWashington, DC, USA; ^3^Department of Neurology, Georgetown UniversityWashington, DC, USA

**Keywords:** risk taking, decision-making, residential choice, aging, computational modeling

## Abstract

As our society becomes more mobile and people reside farther away from their immediate families, competent decision-making has become critical for the older adults wishing to maintain their independence. However, very little is known about the relationship between residential choice and decision-making. Here we use the Balloon Analog Risk Task (BART) to examine risk-taking in two samples of older adults, one living in a retirement community and another living independently. We also used a cognitive model to gain insight into the cognitive factors underlying decision-making in these groups. We found that older adults living in a retirement community were more risk averse than their independent counterparts. Furthermore, this difference appeared to be motivated by group differences in initial perception of risk. This study suggests an intriguing difference between these two residential groups, and also points to the utility of using laboratory methods in research on real-world problems.

## Introduction

Demographic and cultural trends are rapidly shifting the face of aging in America and around the world (The United Nations, 2002; Shrestha and Heisler, 2011). As a consequence, there is a growing emphasis on personal responsibility and independent living, placing older adults in the position of making an increasing number of consequential decisions. This has raised concerns and spurred research about how older adults make complex decisions about finances and health care (Finucane et al., 2002, 2005; Moye and Marson, 2007; Weierich et al., 2011; Li et al., 2013). However, recent studies have shown that age-related differences in risk-taking can depend upon the domain in which decisions are made (Rolison et al., 2014), and one under-studied area is how risk-taking is related to multi-domain decisions. The choice of whether to live independently or in a retirement community is one example of a multi-domain decision, as it often involves consideration of financial, health and social factors (Mack et al., 1997). Thus, as a first step in addressing this gap in research, this study seeks to examine the relationship between risk-taking, cognitive abilities, and residential arrangements.

One of the most commonly used laboratory tasks to measure risk-taking is the Balloon Analog Risk Task (BART; Lejuez et al., 2002). In this task, participants pump up a simulated balloon, earning points for each pump they make. However, if they pump too much, the balloon explodes and all accumulated points are lost. Thus, this task simulates many instances of complex real-life risk-taking behavior in that risk-taking is rewarded up to a point, but further risk-taking is penalized. Performance on the BART has been shown to be a strong predictor of real-life risk-taking behavior in a variety of clinical and developmental populations (Lejuez et al., 2003a,b; Aklin et al., 2005; Hunt et al., 2005; Hopko et al., 2006; Bornovalova et al., 2009), including older adults (Henninger et al., 2010; Cavanagh et al., 2012; Rolison et al., 2012), thereby demonstrating its ecological validity. In fact, the BART is sometimes a better predictor of real-life risk-taking behavior than demographic and self-report measures (Lejuez et al., 2002). While initial uses of the BART were very informative about the risk-taking tendencies of a variety of populations, they did not directly focus on disentangling the cognitive factors that may underlie risk-taking behavior. To accomplish this, recent work has adopted a cognitive modeling approach.

The use of cognitive models is gaining popularity in cognitive science, in part because of their usefulness in formalizing conceptual understanding into testable models of cognition (Busemeyer and Diederich, 2010), often exposing underlying variables that influence behavior. Several cognitive models of behavior on the BART have been developed and tested, providing insight into the cognitive processes underlying risk-taking behavior. The model that has been shown to best predict behavior, Wallsten et al.'s Model 3 (2005), makes three key assumptions. First, it assumes that participants begin the task with certain expectations and a risk-taking propensity, which influences their initial perception of risk and their confidence in this estimate. Second, it assumes that decision makers adjust this risk estimate based on their experience with winning or losing on each balloon, and third, it assumes that people believe the probability of the balloon bursting on each trial (or balloon) is fixed across pump opportunities. Additionally, many of the model parameters have been associated with real-world risk taking, including drug use, unprotected sex, and stealing (Wallsten et al., 2005). Thus, computational models of the BART are useful for informing our understanding of risk-taking and for identifying the factors that influence risk-taking in a variety of populations.

To date, only a handful of studies have used the BART to examine risk-taking in older adults. One study has shown an inverse relationship between risk-taking and age, with younger age being associated with greater risk-taking behavior (Henninger et al., 2010). However, other studies have found that age differences in risk-taking only exist under conditions of high risk (Cavanagh et al., 2012) or with experience (Rolison et al., 2012). Furthermore, using Wallsten et al.'s Model 3, Rolison et al. (2012) found a significant relationship between age and initial beliefs, such that older adults were initially more risk-avoidant than younger adults, and they had less confidence in these initial estimates than their younger counterparts. However, it is important to note that these age differences disappeared with experience (Rolison et al., 2012). These studies indicate that the BART is useful for assessing risk-taking in older adults. One open question, however, is whether important decisions that have *already* been made by older adults (e.g., whether to live independently or in a senior community) are related to their risk-taking propensity.

The goal of this study is to examine the relationship between risk-taking behavior and residential choice in older adults. We examined risk-taking behavior using the BART in two convenience samples of older adults, one residing in a retirement community and another living independently. We then examined the cognitive constructs underlying risk-taking by fitting a computational model to their data to see if there were systematic differences between the two groups in terms of underlying cognitive factors influencing risk-taking.

## Methods

### Participants

Forty-six older adults volunteered to participate. Twenty-three were *Independent* living volunteers (79.87 ± 5.87 years old), a subsample of 64 older adults who responded to advertisements placed in regional newspapers for another study. Twenty-three were volunteers residing in a retirement community (*Retirement*; 79.91 ± 9.72 years old) who responded to advertisements placed within the community. All received gift certificates for participating. As shown in Table 1, these groups were matched in terms of measured demographic and neuropsychological characteristics, as none of the differences were significant.

**Table 1:** **Mean values (with standard deviation in parentheses) of participant characteristics**.

**Variable**	**Independent**	**Retirement**
Gender	12 Female, 11 Male	17 Female, 6 Male
Age (in years)^a^	79.87 (5.87)	79.91 (9.72)
WMS-III Digit Span Backwards	6.22 (1.81)	6.70 (2.67)
NAART35 Vocabulary^b^^,^^c^	9.70 (6.87)	13.43 (9.33)
*N*	23	23

*Independent, Independent older adults; Retirement, Retirement community older adults; WMS-III, Wechsler Memory Scale—Third Edition (Wechsler, 1997); NAART35, Short Version of North American Adult Reading Test (Uttl, 2002)*.

a *Age was not recorded for one Retirement participant*.

b *NAART Vocabulary is scored such that higher scores reflect poorer performance. For all other neuropsychological tests, higher scores indicate better performance*.

c *NAART was not administered to three participants in the Independent group*.

### Balloon analog risk task (BART)

Risky behavior was measured using 30 trials of a computerized version of the BART (Lejuez et al., 2002) programmed in E-Prime 2 (McFarlane, 2008). On each trial, participants were shown an image of a balloon and were given the option to inflate the balloon by pressing the “p” key on the keyboard or to cash out for that balloon by pressing the “s” key. For each pump, the balloon increased in size, and 10 points were added to a temporary bank that was displayed on the bottom right of the screen. When a participant chose to cash out, a screen appeared telling them how many points they had earned for that trial; all points in the temporary bank were then added to their Total Points. If the balloon exploded before a participant cashed out, however, a screen appeared indicating that the balloon had exploded, and all the points in the temporary bank were lost. Thus, the Total Points box kept track of all points earned across trials and it remained on the screen throughout the task. The probability that the balloon would explode increased linearly from 1/128 on the first pump to 1/1 on the 128th pump, exploding on average at 64 pumps. Optimal behavior is to pump 64 times on each trial, although research with younger adults suggests participants typically pump about half this amount (Lejuez et al., 2002; blue balloons).

### Procedures

All participants gave informed consent and completed the BART as part of a larger study. *Independent* participants were tested on the first day of a 2-day experimental protocol in the Cognitive Aging Laboratory at The Catholic University of America. *Retirement* participants were tested on the second day of a 2-day experimental protocol at Friendship Terrace Senior Living Community in Washington, DC. In both protocols, participants completed the BART near the end of the testing session for that day. The Institutional Review Boards at The Catholic University of America and at Georgetown University approved the *Independent* and *Retirement* experimental protocols, respectively. In both protocols, participants also completed a short biographical questionnaire, the Digit Span Backward (DSB; Wechsler, 1997) and North American Adult Reading Test (NAART; Uttl, 2002).

All participants were seated in front of a computer in a quiet room to complete the BART. Instructions, which were presented on the screen, explained that their task was to inflate the balloon as large as possible without it exploding. They were told they would receive points for each successful pump, and that if the balloon exploded they would lose all the points for that trial. Participants completed one practice trial, or balloon, before moving on to the 30 experimental trials, or 30 balloons.

## Results

### Risk taking

We quantified risk-taking behavior on the BART using three measures: *adjusted number of pumps, total points earned*, and *number of explosions*. The most commonly reported measure of risk-taking behavior on the BART is the *adjusted number of pumps*, or the average number of pumps across trials, excluding the trials on which the balloon exploded. Thus, a larger value for adjusted number of pumps represents greater risk-taking behavior that is unbiased by the exploded balloons. We adopted this adjusted measure, originally described by Lejuez et al. (2002), because the number of pumps on the explode trials are limited by the explosion (i.e., if the balloon had not exploded, the participant might have continued to pump). For example, if the balloon explodes after 10 pumps, then we have no idea how many times the participants *would have* pumped if they had been allowed to continue pumping past 10 pumps. Therefore, excluding explosion trials gives a better estimate of risk-taking behavior.

**Figure 1:**
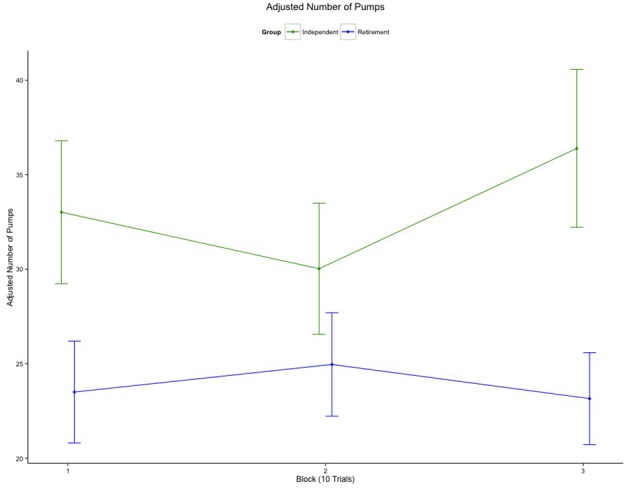
**Risk taking with experience by group**. Average number of pumps per block on trials where the balloon did not explode, separated by group.

Using this measure, there was a significant difference between the two residential groups in terms of risk-taking behavior with those in the Independent group pumping more (*M* = 34.41) than the Retirement group (*M* = 23.56), *t*_(44)_ = 2.76, *p* = 0.008. To determine whether or not risk taking changed with experience, we binned the data into three, 10-trial blocks. As can be seen in Figure 1, the main effect of group remained significant, *F*_(1, 43)_ = 4.99, *p* = 0.031, $\eta_{g}^{2}$ = 0.084, and although there was also a marginal interaction between group and block, *F*_(2, 86)_ = 2.41, *p* = 0.096, the groups differed from each other on both the first block, *t*_(44)_ = 2.27, *p* = 0.032, and the last, *t*_(44)_ = 2.71, *p* = 0.010. Thus, the most sensitive measure of risk taking suggests there is a consistent group difference in risk taking throughout the task.

A similar pattern was seen when we examined the *total points earned*, with participants in the Independent group earning more points (*M* = 6551) than those in the Retirement group (*M* = 5249), *t*_(44)_ = 2.031, *p* = 0.048. Although participants in the Independent (*M* = 7.70) had a greater *number of explosions* than those in the Retirement group (*M* = 6.74), this difference failed to reach significance (*p* > 0.10). However, this measure of risk taking is likely less sensitive than the other risk-taking measures due to its restricted range (0–30). Thus, there were group differences in risk-taking behavior in two out of three measures and this group difference appeared early in the task.

Furthermore, there were no significant correlations between measured individual characteristics and risk-taking behavior on the BART (Table 2, *p*s > 0.10). Collectively, these analyses suggest that those in the Independent group took more risks than those in the Retirement group and that this difference cannot be attributed to group differences in any of the individual characteristics assessed.

**Table 2:** **Correlations between individual characteristics, performance, and model parameters for the Balloon Analog Risk Task (BART) across both groups**.

**Characteristics**	**Risk taking**			**Model parameters**			
	**AP**	**P**	**E**	***β***	***γ*^+^**	***E*(*q*_1_)^a^**	**var(${\hat{q}}_{1}$)**
Gender^b^	−0.122	0.047	0.090	−0.302^*^	0.145	−0.169	0.136
Age	−0.008	0.055	−0.049	0.063	0.034	−0.290^†^	0.314^*^
Education	−0.058	−0.003	−0.114	0.076	−0.122	0.201	−0.119
NAART35 vocabulary^c^	−0.013	−0.031	0.053	−0.115	0.069	−0.156	0.064
WMS-III digit span backwards	0.106	−0.017	0.129	0.338^*^	−0.136	0.024	0.013

*AP, Adjusted Number of Pumps on BART (Lejuez et al., 2002); P, Total Points on BART; E, Number of Explosions on BART; WMS-III, Wechsler Memory Scale—Third Edition (Wechsler, 1997); NAART35, Short Version of North American Adult Reading Test (Uttl, 2002)*.

a *Initial risk perception is calculated such that higher values denote lower initial risk perception*.

b *Gender was encoded as female = 1, male = 0*.

c *NAART Vocabulary is scored such that higher scores are related to poorer performance. For all other neuropsychological tests, higher scores indicate better performance*.

† *p < 0.10*,

* *p < 0.05*.

### Model parameter estimates

Next, underlying cognitive variables that may influence risk-taking behavior were examined using computational modeling. A cognitive model with four free parameters was fit to each individual's performance on the BART (Wallsten et al., 2005; Cavanagh et al., 2012; Rolison et al., 2012) to provide a theory-based assessment of the cognitive factors underlying risk taking. This model assumes that participants begin the task with an initial belief about the probability that the balloon will explode, and then adjust this prior estimate after each trial. This probability for any given trial, *k*, is estimated with the following equation:

$$p_{k}^{belief}=1-\frac{\alpha_{0}+\sum_{K=0}^{k-1}n_{K}^{succes}}{\mu_{0}+\sum_{K}^{k-1}n_{K}^{pumps}}with\;\alpha <\mu$$

where the prior belief (1 − α_0_/μ_0_) is updated by the adding the ratio of the number of successful pumps thus far ($\sum_{K=0}^{k-1}n_{K}^{succes}$) to the total number of pumps thus far ($\sum_{K}^{k-1}n_{K}^{pumps}$). Next, this probability is used to estimate the optimal number of pumps, ω_*k*_,

$$\begin{array}{l} \omega_{k}=\frac{{-\gamma }^{+}}{ln\left(1-p_{k}^{belief}\right)}with\;\gamma^{+}\geq 0 \end{array}$$

such that increasing *reward sensitivity* (γ^+^) inflates the estimate of the optimal number of pumps. Finally, *behavioral consistency* (β), or the degree to which a participant uses this estimate on a given pumping opportunity *l* for trial *k*, is estimated such that:

$$\begin{array}{l} p_{kl}^{pump}=\frac{1}{1+e^{\beta \left(l-\omega_{k}\right)}}with\;\beta \geq 0 \end{array}$$

For interpretation, instead of discussing the prior belief that the balloon will explode, (1 − α_0_/μ_0_), researchers typically calculate the probability the balloon will not explode, because this is the probability that the participant will pump. This is known as *initial risk perception* (*E*(*q*_1_)), where *q*_1_ = α_0_/μ_0_. How much this perception of risk varies with experience is known as *confidence in the initial risk perception* (var(${\hat{q}}_{1}$)), and is calculated as follows:

$$\begin{array}{l} Var\left(q_{1}\right)\;=\;\frac{\alpha_{0}\left(\mu_{0}-\alpha_{0}\right)}{\mu_{0}^{2}\left(\mu_{0}+1\right)} \end{array}$$

To summarize, two parameters are directly estimated in the model and describe the consistency of the participant's responses (*behavioral consistency*, β) and how a participant adjusts his/her estimate of risk based on experience in the task (*reward sensitivity*, γ^+^). For each of these parameters, higher values indicate greater consistency or sensitivity, respectively. The other parameters, *initial risk perception* (*E*(*q*_1_)) and *confidence in that perception* (var(${\hat{q}}_{1}$)) are derived from model parameters (α and μ) and estimate each participant's initial perception of risk prior to beginning the task. For *initial risk perception* (*E*(*q*_1_)), higher values indicate a greater initial belief that the balloon will not explode, or lower perception of risk. For the other derived parameter, *confidence in the initial risk estimate* (var(${\hat{q}}_{1}$)), higher values indicate greater uncertainty in the initial estimate of risk.

This cognitive model was compared to a baseline model that assumes the participant is equally likely to pump on each opportunity for each balloon (Wallsten et al., 2005). Model fit was evaluated for each participant by calculating the Akaike Information Criterion (AIC) for each model, which penalizes more complex models based on their complexity (Akaike, 1974). Lower AIC values mean better model fit. For all participants, this cognitive model was a better fit than the baseline model. Furthermore, the model fit did not vary significantly between the Independent (*M* = 149) and Retirement (*M* = 156) groups, *t*_(44)_ = −0.83, *p* = 0.411.

### Individual characteristics and risk

First, we examined the Pearson bivariate correlations between the model parameters and all measured individual characteristics. As can be seen in Table 2, across both groups there was a marginally significant relationship between age and the *initial perception of risk, E(q*_1_), *r*_(43)_ = −0.290, *p* = 0.054, such that older individuals had higher initial perceptions of risk. In addition, there was a significant relationship between age and *confidence in the initial risk estimate* var(${\hat{q}}_{1}$), *r*_(43)_ = 0.314, *p* = 0.036, such that older individuals were less confident in this initial estimate. There was also a significant relationship between backward digit span and *behavioral consistency* β, *r*_(44)_ = 0.338, *p* = 0.022; those with larger working memory demonstrated greater *behavioral consistency*. Finally, the relationship between gender and *behavioral consistency* was also significant, *r*_(44)_ = −0.302, *p* = 0.042, with males showing more behavioral consistency than females. There were no other significant relationships between model parameters and the measured individual characteristics. Thus, the *initial perception of risk* and *confidence in it* were related to age, while *behavioral consistency* was linked to working memory and gender. For this reason, we included these factors as covariates in our analysis of group differences.

### Residential group and risk

Although there were no significant group effects for *behavioral consistency* (β), *reward sensitivity* (γ^+^), or *confidence in the initial risk estimate* var(${\hat{q}}_{1}$), a One-Way ANCOVA on *initial perception of risk E(q*_1_), controlling for age, gender and working memory, revealed a marginal main effect of Group, *F*_(1, 40)_ = 3.84, *p* = 0.057, $\eta_{p}^{2}$ = 0.088, with individuals in the Independent group (*M* = 0.99, *SD* = 0.025) perceiving less risk than those in the Retirement group (*M* = 0.98, *SD* = 0.025). Similar to the risk-taking behaviors, these modeling results suggest that those in the Independent group were more willing to take risks because their initial perception of risk was lower than their Retirement counterparts.

## Discussion

This study examined the relationship between risk-taking behavior and residential choice by administering the BART to two samples of older adults, one residing in a retirement community and another living independently. Our primary finding was a significant difference between the two groups in risk-taking behavior, with those residing independently taking more risks on the BART than those living in a retirement community. Furthermore, block-level analyses and computational modeling results suggest that those residing independently had lower initial perceptions of risk. Collapsing across groups, we also found a relationship between age and initial perceptions of risk, with older individuals having marginally higher baseline perceptions of risk and significantly less confidence in these estimates.

To our knowledge, the finding that a group of older adults living in a retirement community exhibited more risk-aversion than a group living independently is novel. This suggests that a relationship between residential choice and risk-taking exists, but the directionality of this relationship is still unknown and cannot be determined from the present data. For example, it is possible that risk-averse individuals choose to live in senior living communities—that is, the relationship we detected could be due to pre-existing group differences between the samples. Indeed, there is evidence to support this interpretation in that qualitative studies have shown that when asked, older adults identify many factors that put their ability to live independently at risk, including the inability to cope with health or cognitive problems, changes to physical or financial security, or loss of social support (Mack et al., 1997). It is possible that these risk factors lead more risk-averse individuals to seek the structured, supportive environment of a retirement community. However, it is also possible that people become more risk averse as a result of living in a retirement community. If this is the case, then it would be important to investigate which cognitive processes and/or propensities are affected by living in these communities, as this has critical implications for maintaining and promoting cognitive wellbeing and functional independence in old age. Longitudinal studies, either examining the residential choices that people with different risk-taking propensities make later in life, investigating if risk-aversion increases when people move into senior living communities, or looking at if risk aversion is positively related to time in a retirement community, could help to clarify the direction of causation. Furthermore, the neuropsychological battery used in this study was limited so it is possible that the two groups differ on some other cognitive ability that was not assessed here.

Our block-level analyses and computational modeling results provide further insight into this group difference. When we examined risk-taking behavior at the beginning, middle and end of the task, we found group differences in risk-taking behavior during the first 10 trials. Furthermore, after controlling for individual factors like age, gender, and working memory, we found a marginally significant group difference in *initial perception of risk* with our computational model. Collectively, these results suggest the group difference in risk taking behavior could be due to group differences in risk perception before even beginning the task. Specifically, those in the Residential group were more likely to believe the balloon would burst. Presumably, this initial perception led them to be more risk-averse in the task. Thus, the modeling results provide additional insight into potential individual characteristics that underlie the group differences in risk taking observed.

The relationship between age and initial perceptions of risk, our third finding, is consistent with previous findings reported in the literature. For instance, Rolison et al. (2012) found a significant relationship between age and prior beliefs of risk in a sample of both younger and older adults. Specifically, they found that older adults were initially more risk averse than their younger counterparts, and that older adults were less confident in their initial estimates than younger adults. The fact that we were able to replicate this relationship within a more restricted, older age range suggests that the relationship between age and initial estimates of risk is robust. Importantly, age-related changes in risk estimates have important implications for policy makers, clinicians, and caregivers seeking to help older adults make residential choices.

## Conclusion

As our aging society becomes more mobile, and thus physically separated from their families, older adults are increasingly being asked to make decisions about housing, finances and health care independently. This study demonstrates the utility of using tools of basic science, both simple laboratory tasks and cognitive modeling, to inform discussions of real-world problems like residential choice in older adults and suggests areas of future research. A better understanding of the psychological dispositions and capabilities of older adults making residential decisions could help clinicians guiding older adults and their families in making these decisions, or facilitate the development of decision-making aids for those faced with this dilemma.

## Funding

This work was supported by the National Institute on Aging under Grants 1RO1AG036863 and F31AG047037.

### Conflict of interest statement

The authors declare that the research was conducted in the absence of any commercial or financial relationships that could be construed as a potential conflict of interest.
